# Cardiac abnormalities 15 years and more after adriamycin therapy in 229 childhood survivors of a solid tumour at the Institut Gustave Roussy

**DOI:** 10.1038/sj.bjc.6601904

**Published:** 2004-05-25

**Authors:** F Pein, O Sakiroglu, M Dahan, J Lebidois, P Merlet, A Shamsaldin, E Villain, F de Vathaire, D Sidi, O Hartmann

**Affiliations:** 1 Department of Paediatric Oncology, Institut Gustave Roussy, rue Camille Desmoulins 39, 94805-Villejuif, Villejuif, France; 2 Service of Cardiology, Hospital Beaujon, Clichy, France; 3 Service of Paediatric Cardiology, Hopital Necker-Enfants Malades, Paris, France; 4 Frederic Joliot Service of Médecine Nucléaire, Centre Hospitalier d'Orsay, Orsay, France; 5 Research Unit in Cancer Epidemiology, Institut Gustave Roussy, Villejuif, France

**Keywords:** childhood cancer, anthracycline, late cardiac toxicity

## Abstract

The purpose of this paper was to determine the cardiac status in children 15 years or more after adriamycin therapy for a solid tumour. Of the 447 pts, 229 pts were fully studied and 218 were not. The following cardiac evaluations were proposed to all the 447 consecutive patients (pts): (1) cardiac Doppler US by one of two expert cardiologists; (2) cardiac rhythm and conduction abnormalities including 24-hour holter ECG; (3) ^131^l-mlBG myocardial scintigraphy; (4) serum brain natriuretic peptide levels at rest; (5) an exercise test with VO_2_ max measurement. The radiation doses delivered to 6 points in the heart were estimated for all patients who had received radiotherapy. Congestive heart failure was diagnosed in 24 of 229 (10%) evaluated pts, with a median interval of 15 years (0.3–24 years) from the first symptom after adriamycin treatment. Among the 205 remaining pts, 13 asymptomatic pts (6%) had severe (*n*=4) (FS<20%) or marked (*n*=9) (20⩽FS<25%) systolic dysfunction. In the 192 others, the median meridional end-systolic wall stress was 91 (53–135) and it exceeded 100 g cm^−2^ in 52 pts. Using a Cox model, only the cumulative dose of adriamycin and the average radiation dose to the heart, were identified as risk factors for a pathological cardiac status. In conclusion, the risk of cardiac failure or severe abnormalities increases with adriamycin treatment, radiotherapy and time since treatment, even after a follow-up of 15 years or more. In our series, after an average follow-up of 18 years, 39% of the children had a severe cardiac dysfunction or major ventricular overload conditions. The risk increases with the dose of adriamycin and radiation received to the heart, without evidence for threshold.

Despite strong evidence for the cardiac toxicity (Von Hoff *et al*, 1979; Grenier and Lipshultz, 1998) of anthracyclines, they continue to be among the most frequently used cytotoxic agents in adult and paediatric oncology more than 30 years after their discovery (Abraham *et al*, 1996; Hortobagyi, 1997). The late cardiac events among long-term childhood leukaemia and cancer survivors were first described in 1991 (Lipshultz *et al*, 1991; Steinherz *et al*, 1991, 1993), then later confirmed by many other teams. It was hypothesised that the loss of myocytes during anthracycline therapy might impair myocardial growth and lead to a gradual increase in left ventricular afterload and sometimes reduced contractility (Lipshultz *et al*, 1991, 1995; Grenier and Lipshultz, 1998). An increased risk was found to be associated with mediastinal radiotherapy and the time since drug administration (Steinherz *et al*, 1991, 1993): the role of pregnancy, delivery, and weight-lifting activities in revealing cardiac deficits (Steinherz *et al*, 1993, 1995) was highlighted. The frequency of cardiac abnormalities according to the anthracycline dose has been correlated with age at treatment, the time elapsed since treatment, and sex (Lipshultz *et al*, 1995; Silber *et al*, 1993; Steinherz *et al*, 1995). More recently, a review listed all the studies in order to identify well-known risk factors for cardiac abnormalities (Kremer *et al*, 2002).

However, to date, no study with such a large series of patients and a minimum follow-up of 15 years, has attempted to undertake a comprehensive evaluation of cardiac parameters with techniques other than Doppler US, and controlled for drugs other than adriamycin and for the radiation dose received by the heart at the time of radiotherapy, whatever the target organ.

## METHODS

### Patients

A total of 447 consecutive patients (pts) who received at least one dose of doxorubicin or daunorubicin (only two pts) between 1968 and 1982 and who were alive at the end of treatment of a solid tumour at the Institut Gustave Roussy (IGR) were selected. Clinical and histopathological characteristics of the cancers, the type of treatment, detailed information on chemotherapy, follow-up data, and medical information about second cancers, were extracted from hospital clinical records.

Information about the vital status and the date and cause of death of each patient was obtained from the National Institute of Statistics and Economic Studies (INSEE). For each dead patient, the initial, immediate, and associated causes of death, coded according to the 9th version of the International Classification of Diseases, were obtained from the Center of Epidemiology on Medical Causes of Death at the National Institute of Health and Medical Research (CépiDc INSERM) for every patient who had died during the 1969–1999 period.

### Cardiac evaluation

A letter was sent to each living patient in order to propose to him the following investigations by an expert team of cardiologists:

1. Cardiac Doppler Ultrasonography by one of two expert cardiologists: measurements of the left ventricular (LV) and derived variables were made from both M-mode and two-dimensional echocardiograms. M-mode measurements were made at the tip of the mitral valve or just below. LV internal dimensions and septal and posterior wall thickness were measured at end diastole and at end systole over an average of up to five cardiac cycles, according to the American Society of Echocardiography recommendations (Schiller *et al*, 1989). Blood pressure was measured with subjects lying supine for at least 15 min in a quiet room before the echocardiogram, using a mercury sphygmomanometer. The three main derived variables used to define cardiac abnormalities were calculated as follows:

- Fractional shortening (FS) (%)=(LVEdD−LVEsD)/LVEdD where LVEdD is the left ventricular end-diastolic diameter, and LVEsD, the left ventricular end-systolic diameter. Normal FS value ⩾28% in healthy adults.
- Ejection fraction (EF) (%)= (LVEdV−LVEsV)/LVEdV where LVEdV is the left ventricular end-diastolic volume, and LVEsV the left ventricular end-systolic volume; the left ventricular volumes were calculated according to the Teichholz equation: *V*= (7/2.4+*D*) × *D*^3^. Normal EF value ⩾60% in healthy adults.
- End-systolic meridional wall stress (ESWS) was calculated using the Sandler–Dodge formula (Sandler and Dodge, 1963) for a thin-walled prolate ellipsoid:

Stress (in kdynes cm^−2^)=(1.333 × SBP × 2*r*)/*h*(2*r*+*h*) where 1.333 is the conversion factor from mmHg to kdynes, SBP is the cuff systolic blood pressure, and *r* (radius; 2*r*=LVEsD) and *h* (thickness) correspond to (systolic septal wall thickness+systolic posterior wall thickness)/2.

One kilodyne (kdyne), i.e. 10^3^ dynes ≈ 1.01972 g force cm^−2^, i.e. ≈1 g cm^−2^

ESWS normal values=68±18 kdynes cm^−2^ as measured in 50 normal subjects (Ganau *et al*, 1990).

2. A 24-hour holter ECG registered on a portable audiotape.

3. ^123^l-mlBG myocardial scintigraphy, calculating the cardiac to mediastinum fixation ratio at the fourth hour (CM4) after an isotope injection. This ratio is a good indicator of the catecholamine uptake by the myocardiac muscle cells. In case of decrease in such a ratio, it can be concluded that the sympathetic myocardium innervation has been decreased, according to the number of muscle cells killed.

4. Serum brain natriuretic peptide (BNP) levels at rest.

5. Exercise test with VO_2_ maximum consumption (VO_2_ max) measurement.

6. Left ventricular diastolic function, measured by the E wave/A wave ratio, which is the ratio between the ‘peak Early diastolic phase filling’ and the ‘Atrial diastolic phase filling’. It measures the left ventricular diastolic dysfunction, characterised by a decrease of the early diastolic filling and an increase of the atrial diastolic filling. Ratios lower than 1.5 are considered as suspicious and ratios lower than 1 as clearly abnormal (Bu'Lock *et al*, 1995).

The presence of a cardiac abnormality was defined as one of the following criteria: either one cardiac failure episode with clinical congestive signs, according to NYHA classification, or, in nonsymptomatic patients, alteration of any of these three parameters: FS<25% and/or EF<50% and/or ESWS>90 g cm^−2^.

### Dosimetry

Radiotherapy data were obtained from technical radiotherapy records by hospital physicists. Among the 447 pts, 245 had received radiotherapy at some site in the body. Whatever the site of the target volume, the mean radiation dose to the heart for each patient who had received external radiotherapy, we used a computer program called ‘Dos_EG’ to calculate these data (Diallo *et al*, 1996; Shamsaldin *et al*, 1998). Six anatomical points for dose estimations were taken into account in the heart, and the mean dose of radiation received to the heart was estimated, for each patient, as the mean dose to these six anatomical sites.

Among the 245 pts who had received external radiotherapy, the mean dose to the heart was 6.7 Gy, but the median dose was only 3.7 Gy (range: 0.001–91). Despite the variations in treatment machines, the mean dose to the heart did not vary according to the calendar period: 6.7 Gy before 1975, 6.7 Gy between 1976 and 1980, and 6.9 Gy after 1981.

### Statistical analysis

The cumulative incidence of cardiac failure was determined using the Kaplan–Meier method, and risk factors for cardiac failure were identified using Cox's proportional hazard regression models (Breslow and Day, 1987). Assessment of relationships between cardiac parameters was conducted using linear regression, after transformation when they were abnormal. As the cardiac status was assessed at a time that we decided (i.e. at the time of the cardiac investigation), it was not possible to ascertain the time of onset of an abnormal cardiac status; therefore, we were unable to study the temporal pattern of the occurrence of this event. Risk factors for a pathological cardiac status were identified via logistic regression models (Breslow and Day, 1980).

## RESULTS

Of the 447 pts, 218 pts did not participate in the study because they refused to participate (*n*=174), were lost to follow-up (*n*=37), or were dead (*n*=7). The causes of death were as follows: Creutzfeldt–Jakob disease (*n*=2), second malignancy (*n*=2), suicide, car crash, and unknown cause (*n*=1).

Of the 229 other pts, 205 were examined according to the previously described protocol, and 24 were not examined but were included in the study because they had experienced a clinical cardiac failure diagnosed by their own cardiologist before their entry in the study. We contacted all these specialists in order to verify this diagnostic of cardiac failure episode with clinical congestive signs according to NYHA classification and to obtain additional information. Table 1

**Table 1:** Study population

			**Examined or known cardiac failure (*n*=229)**		
	**Whole population (*n*=447)**	**Not examined and not known cardiac failure (*n*=218)**	**Cardiac failure (*n*=24)**	**Asymptomatic patients examined (*n*=205)**	***P*-value**^a^
Date of first adriamycin dose	1978 (1968–1982)	1978 (1972–1983)	1978 (1973–1985)	1978 (1968–1984)	NS
Sex (M/F)	278/169	145/73	14/10	119/86	0.1
					
*First cancer type*					
Unknown	3	1	0	2	
Lymphoma	138	67	3	68	
Neuroblastoma	64	25	6	33	
Nephroblastoma	56	21	8	27	NS
Soft-tissue sarcoma	91	54	1	36	
Osteosarcoma	15	5	1	9	
Ewing sarcoma and PNET	33	19	2	12	
Hepatoblastoma	8	4	0	4	
UCNT and other carcinoma	7	2	1	4	
Others	32	20	2	10	
					
*First cancer site*					
Unknown	2	1	0	1	
Brain and spinal axis	5	2	1	2	
Skull, face and orbit	51	28	0	23	
Cervical	51	20	1	30	
Thorax and mediastinum	49	20	5	24	
Retroperitoneum	126	56	11	59	
Abdominal cavity	45	22	0	23	
Pelvis	52	32	3	17	
Rachis	17	12	1	4	
Limbs	49	25	2	22	
					
Age at first anthracycline dose (min–max)	6.2 (0–21)	6.9 (0–16)	4.8 (1–13)	5.7 (0–21)	0.005
Total anthracycline dose: mean (min–max) (mg m^−2^)	344 (40–600)	347 (60–600)	412 (180–600)	333 (40–600)	0.05
Radiotherapy: *n* (%)	245 (55%)	125 (57%)	14 (58%)	106 (52%)	0.3
Radiation dose to the heart: mean (min–max) (Gy)	6.7 (0–91)	5.2 (0–35)	13.4 (0–46)	7.7 (0–91)	0.3
Maximal radiation dose at a point in the heart: mean (min–max) (Gy)	31.3 (0–125)	32.5 (0–102)	37.3 (0–745)	31.5 (0–125)	0.5

a Comparison between the 205 pts not examined and the others.

presents the general characteristics of the 447 pts and of their chemotherapy treatment.

### Cardiac failure

The 24 pts who experienced cardiac failure were younger at the time of the first adriamycin treatment than the 218 patients who were not examined and the 205 asymptomatic patients who were examined (Table 1). On the whole, patients who experienced cardiac failure had received a higher cumulative adriamycin dose (*P*=0.01) and a higher radiation dose to the heart (*P*=0.005) than patients who had not.

**Figure 1:**
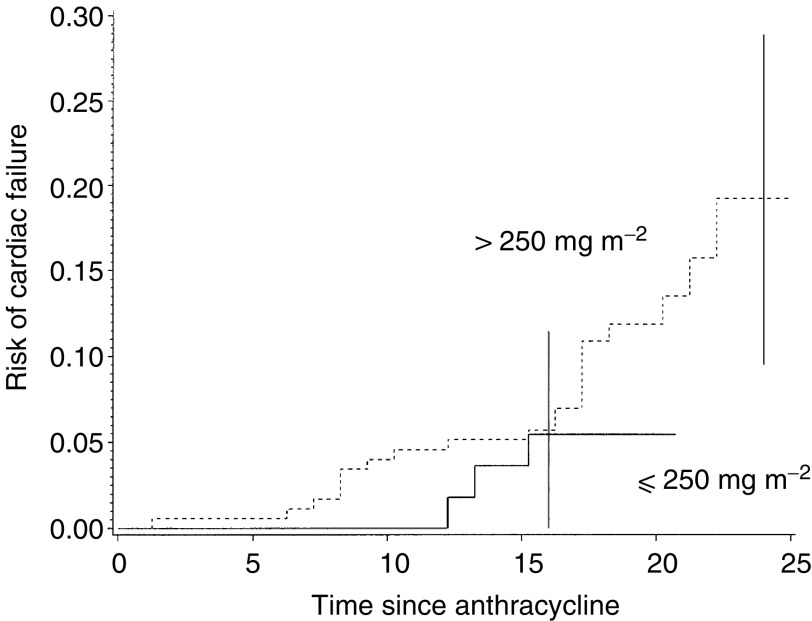
Risk of cardiac failure and time since anthracycline exposure.

As shown in Figure 1

, the risk of cardiac failure increased regularly with time since the first adriamycin treatment and attained 19% (95% Cl: 9–29%) 25 years later, for patients who had received more than 250 mg m^−2^. Both adriamycin treatment and the average dose of radiation to the heart played a significant independent role in the risk of cardiac failure (Table 2

**Table 2:** Prognostic factors for cardiac failure and cardiac disorder in 229 patients

	**Cardiac failure (***n***=24)**		**Cardiac disorder (***n***=89)^a^**	
**Parameters**	**Relative risk (95% CI)**	***P*-value**	**Relative risk (95% CI)**	***P*-value**
*Gender*				
Male	1^b^		1^b^	
Female	1.03 (0.45–2.37)	NS	1.41 (0.8–2.6)	NS
Age at first treatment				
⩾8 years	1^b^		1^b^	
0–7 yrs	2.63 (0.87–7.96)	0.08	3.21 (1.63–6.34)	<0.001
Cumulative anthracycline dose (RR at 100 mg m^−2^)	1.99 (1.30–3.01)	<0.005	1.60 (1.22–2.09)	<0.001
Average radiation dose (RR at Gy to the heart)	1.12 (0.95–1.29)	<0.01	1.25 (0.99–1.50)	<0.001

a At least one of the following criteria: cardiac failure, FS<25, ES<50, or ESWS>100.

b Reference category.

). We evidenced a near significant reduction in the risk for the 8 years or older age category at the time of the first adriamycin treatment (Table 2), but we were not able to evidence a threshold for a younger age. A detailed analysis of the dose–response relationship was not possible because of the small number of events. Compared to patients who had received 250 mg m^−2^ or less, those who had received 250–400 mg m^−2^ experienced a relative risk of cardiac failure of 1.93 (95% Cl: 0.49–5.8), and those who had received 400 mg m^−2^ or more, a relative risk of 4.92 (95% Cl: 1.28–18.9). There was a trend (*P*=0.1) towards an interaction for a greater increase in the risk of cardiac failure per amount of the adriamycin cumulative dose among boys (RR for 100 mg m^−2^=2.88, 95% Cl: 1.44–5.73) than among girls (RR for 100 mg m^−2^=1.70, 95% Cl: 0.78–3.69).

We were not able to evidence an excess risk for patients who had received less than 5 Gy of average radiation dose to the heart (RR=0.45, 95% Cl: 0.12–1.68), as compared to patients who had not received radiotherapy. In contrast, those who had received an average dose of between 5 and 20 Gy had a relative risk of cardiac failure of 2.52 (95% Cl: 0.96–6.60), and those who had received 20 Gy or more, a relative risk of 5.65 (95% Cl: 1.45–22.0). In contrast to that observed for adriamycin therapy, the increase in the risk per average radiation dose to the heart seemed higher (*P*=0.08) among girls (RR at 1 Gy=1.18, 95% Cl: 0.91–1.44) than among boys (RR at 1 Gy=1.09, 95% Cl: 0.93–1.25). Nevertheless, such an interaction was not found when considering the maximal dose received at any site of the heart.

We were able to evidence neither synergy between the effects of adriamycin therapy and the radiation dose (*P*=0.8) nor an interaction. Nevertheless, the power of such a test was very low. Among the 34 pts who had received 250 mg m^−2^ or more of adriamycin and 5 Gy or more to the heart, the 25-year risk of cardiac failure (*n*=8) was estimated to be 34% (95% Cl: 5–64%). Of the 24 pts who experienced a cardiac failure, 11 received more than 5 Gy to the heart or more, 21 received more than 250 mg m^−2^ of adriamycin, and eight pts received both 5 Gy or more to the heart and more than 250 mg m^−2^ of adriamycin. Of these 24 pts, two died before transplantation and three after.

### Asymptomatic cardiac abnormalities

Among the 205 asymptomatic patients examined, four had severe (FS<20%), and nine had marked (20⩽FS<25%) systolic dysfunction, requiring treatment and a strict cardiac follow-up (Table 3

**Table 3:** Cardiac parameters in the 205 asymptomatic patients examined according to sex

	**Whole cohort**	**Sex**		
**Cardiac parameters**	**(205)**	**Male (119)**	**Female (86)**	***P*-value**
*FS*				
Mean (s.d.)	33 (5.7)	32 (5.7)	33 (5.8)	0.08
Median (min–max)	32 (13–47)	32 (14–47)	33 (13–46)	
<20	4	2	2	
[20–25[	9	8	1	
[25+	192	108	82	
				
*EF*				
Mean (s.d.)	60 (8)	59 (8)	61 (8)	0.04
Median (min–max)	61 (29–78)	59 (29–78)	62 (29–78)	
<40	5	2	3	
[40–50 [	12	9	3	
[50+	188	108	80	
				
*ESWS*				
Mean (s.d.)	93 (18)	90 (16)	96 (19)	0.06
Median (min–max)	91 (56–178)	91 (56–147)	94 (66–178)	
<90	95	59	36	
[90-100 [	49	29	20	
[100–125]	49	27	22	
>125	12	4	8	
				
*ESWS/VTS ratio*				
Mean (s.d.)	2.3 (0.91)	2.0 (0.68)	2.7 (1.0)	
Median (min–max)	2.2 (0.66–9.5)	1.9 (0.66–4.5)	2.6 (1.1–9.5)	<0.001
<1.5	28	25	3	
[1.5–1.75 [	32	27	5	
[1.75–2 [	17	10	7	
>2	128	57	71	
				
*Cardiac MIBG: CM4 (199 pts studied)*				
Mean (s.d.)	1.9 (0.25)	1.9 (0.25)	1.9 (0.26)	NS
Median (min–max)	1.9 (1.2–2.6)	1.9 (1.2–2.6)	1.9 (1.2–2.6)	
<1.7	33	19	14	
[1.7–2 [	97	54	43	
[2+	69	43	26	
				
*BNP (154 pts studied)*				
Mean (s.d.)	17 (12)	17 (12)	18 (13)	NS
Median (min–max)	19 (0–77)	17 (0–42)	20 (0.2–77)	
<17	73	47	26	
[17–25 [	35	15	20	
[25 30 [	24	14	10	
[30+	22	15	7	
				
*Wave E/Wave A (202 pts studied)*				
Mean (s.d.)	1.9 (0.64)	1.8 (0.63)	1.9 (0.64)	0.1
Median (min–max)	1.7 (0.71–3.7)	1.7 (0.79–3.7)	1.8 (0.71–3.7)	
<1	5	4	1	
[1–1.5[	57	37	20	
[1.5+	140	75	65	

). This pattern was strongly linked to the cumulative adriamycin dose (*P*<0.0001), after adjustment on sex, the type of first cancer, radiation dose to the heart, age at first adriamycin treatment and the interval since treatment (Table 4

**Table 4:** Cardiac parameters in the 205 asymptomatic patients examined according to cumulative anthracycline dose (mg m^−2^)

	**Cumulative anthracycline dose (mg m^−2^)**				**Crude**	**Adjusteda**
**Cardiac parameters**	**⩽150**	**151–250**	**251–400**	**>400**	**Relationship: ***P***-value**	**Relationships: ***P***-value**
*FS*						
Mean (s.e.m.)	35 (0.9)	34 (0.8)	33 (0.6)	30 (0.9)		
Median (min–max)	36 (27–42)	33 (24–42)	32 (20–47)	30 (13–43)		
<20	0	0	0	4		
[20–25 [	0	1	6	2	<0.0001	<0.0001
[25+	20	30	87	55		
						
*EF*						
Mean (s.e.m.)	64 (1.1)	62 (1.0)	61 (0.8)	57 (1.3)		
Median (min–max)	66 (53–73)	62 (47–73)	61 (41–78)	57 (29–74)		
<40	0	0	0	5		
[40–50 [	0	2	6	4	<0.01	<0.01
[50+	20	29	86	53		
						
*ESWS*						
Mean (s.e.m.)	87 (2.7)	90 (2.1)	92 (1.7)	97 (2.9)		
Median (min–max)	85 (70–119)	92 (66–110)	91 (56–135)	94 (62–178)		
<90	13	15	45	22		
[ 90–100 [	4	9	16	20	<0.01	<0.01
[100–125]	2	7	29	11		
>125	0	0	3	9		
						
*ESWS/VTS ratio*						
Mean (s.e.m.)	2.7 (0.17)	2.3 (0.13)	2.3 (0.08)	2.2 (0.15)		
Median (min–max)	2.6 (1.2–4.0)	2.2 (1.4–3.9)	2.2 (1.2–4.8)	1.9 (0.7–9.5)		
<1.5	1	4	11	12		
[1.5–1.75 [	0	6	13	13		
[1.75–2 [	1	1	6	9	<0.001	0.03
<2	17	20	63	28		
						
*Cardiac MIBG: CM4 (199 pts studied)*						
Mean (s.e.m.)	2.0 (0.05)	1.9 (0.06)	1.9 (0.03)	1.9 (0.03)		
Median (min–max)	2.1 (1.6–2.3)	1.8 (1.3–2.6)	1.9 (1.2–2.5)	1.9 (1.2–2.6)		
<1.7	3	7	11	11		
[1.7–2 [	6	13	48	31	NS	0.01
[2+	10	10	29	20		
						
*BNP (154 pts studied)*						
Mean (s.e.m.)	20 (3.1)	17 (2.2)	17 (1.4)	20 (3.1)		
Median (min–max)	24 (1.7–38)	20 (0.3–36)	18 (0–39)	17 (0.2–78)		
<17	6	11	33	23		
[17–25[	3	9	13	10		
[25 30 [	4	3	12	5	NS	NS
[30+	3	2	10	7		
						
*Wave E/Wave A (202 pts studied)*						
Mean (s.e.m.)	1.9 (0.1)	1.8 (0.1)	1.8 (0.07)	1.9 (0.09)		
Median (min–max)	1.8 (10–3.0)	1.7 (0.9–3.3)	1.7 (0.8–3.7)	1.8 (0.7–3.7)		
<1	0	1	1	3		
[ 1–1.5[	4	10	28	15	NS	NS
[1.5+	16	20	60	44		

^a^Adjusted on sex, type of first cancer, radiation dose to the heart, age at administration, and interval between treatment of first cancer and cardiac examination.

). Similarly, the ejection fraction was significantly depressed in 17 pts, including the 13 who had severe (*n*=4) or marked (*n*=9) systolic dysfunction according to the FS criteria (*P*-value <0.01) for the adjusted relationship with adriamycin therapy.

**Figure 2:**
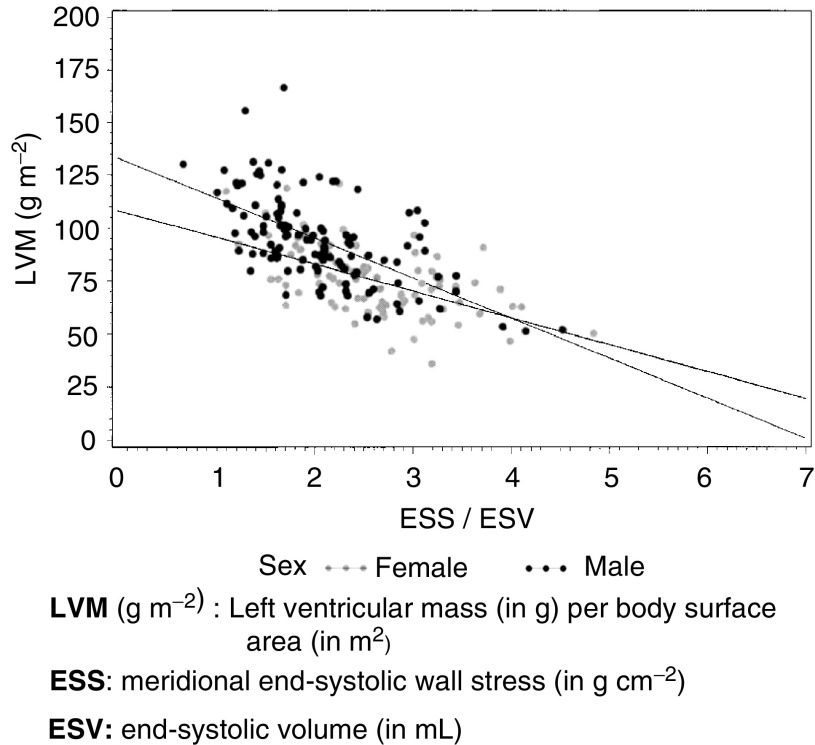
Pressure to volume ratio, according to corrected left ventricular mass in female and male.

In terms of afterload, a total of 61 pts had an ESWS value exceeding 100 g cm^−2^, of whom 52 did not present a severe or marked systolic dysfunction, reflecting an abnormal load condition likely to give rise to systolic function failure due to the thickness of the ventricular wall. Furthermore, the ESWS/end-systolic volume (ESV) ratio, which reflects ventricular contractility, was lower than 1.75 in 59 patients. The ESWS/ESV to left ventricular mass (corrected by the body surface area) ratio, appears nonsignificantly different in boys when compared to girls (Figure 2

).

A total of 65 pts were found to have asymptomatic cardiac disease (FS<25, ES<50, ESWS>100).

Cardiac I^131^ MIBG uptake decreased with higher adriamycin doses, but this was not related to the pathological status, defined as a ratio below 1.7 (Table 4). In all, 15% of the patients with asymptomatic cardiac abnormalities had a CM4 level below 1.7, as compared to 19% of the others (*P*=0.5).

Adriamycin treatment was not found to significantly modify the serum BNP level (Table 4), and the 65 pts with asymptomatic cardiac abnormalities had a serum BNP level similar to that of the other patients (*P*=0.8).

Adriamycin chemotherapy was not found to significantly modify the E wave/A wave ratio (Table 4). In all, 34% of patients with asymptomatic cardiac abnormalities had an E/A ratio below 1.5, as compared to 29% of the others (*P*=0.5).

### All cardiac diseases

A total of 89 patients were identified as having cardiac disease, that is, a severe pathological cardiac status (FS<25%, EF<50%, or ESWS>100 g cm^−2^) or cardiac failure.

The risk of cardiac disease decreased with an older age at the time of cancer treatment, and increased with the total adriamycin dose (Table 5

**Table 5:** Dose–response relationship between the cumulative anthracycline dose and the occurrence of a cardiac abnormality in the 229 patients with known cardiac status

	**Cardiac abnormality^a^**		
**Parameters**	**% (nb/N)**	**Relative risk^b^ and 95% CI**	***P*-value**
Cumulative anthracycline dose (mg m^−2^)			
0–150	10% (2/20)	1^c^	
>150–250	29% (10/34)	2.0 (0.44–9.5)	
>250–400	43% (43/99)	4.0 (0.95–17)	<0.001
>400	45% (34/76)	3.3 (0.78–14)	

a At least one of the following criteria: cardiac failure, FS<25, ES<50, or ESWS>100.

b Adjusted for age at treatment of first cancer, sex, and average radiation dose received at 6 points in the heart, and stratified on the site of the cancer.

c Reference category.

) and with the average dose of radiation delivered to the heart (Table 6

**Table 6:** Dose–response relationship between the average radiation dose to the heart and the occurrence of a cardiac abnormality in 229 patients with known cardiac status

	**Cardiac abnormality^a^**		
**Parameters**	**% (nb/N)**	**Relative risk^b^ and 95% CI**	***P*-value**
*Average radiation dose*			
0 (no radiotherapy)	29% (32/109)	1^c^	
>0–5 Gy	37% (25/68)	1.63 (0.82–3.26)	
>5–20 Gy	63% (26/41)	6.48 (2.76–15.20)	<0.001
>20 Gy	55% (6/11)	4.40 (1.11–17.48)	

a At least one of the following criteria: cardiac failure, FS<25, ES<50, or ESWS>100.

b Adjusted for age at treatment of 1st cancer, sex, and cumulative anthracyline dose, and stratified on the site of the cancer.

c Reference category.

).

#### Adriamycin cumulative dose

There was no apparent threshold for the relationship between the adriamycin dose and the risk of cardiac disease (Tables 2 and 5). The dose–response relation was found to be very similar in boys and girls (*P*-value for an interaction with the sex of the patients: *P*=0.9). Similarly, this relation did not vary according to the age at treatment of the first cancer either with the interval between treatment of the first cancer and the time when cardiac parameters were measured or with cardiac failure.

#### Radiation therapy dose exposure

The risk of cardiac disease was found to be 4.40-fold (95% Cl: 1.0–17. 5) higher for patients who had received more than 20 Gy to the heart than among those who had not received radiotherapy (Table 6). In a manner similar to that found for cardiac failure, we evidenced an interaction between sex and the radiation dose: the excess risk per radiation dose unit was found to be about three-fold higher among girls than boys (*P*=0.05). Greater sensitivity of girls to radiation explained almost all the excess risk observed among girls. When the gender difference in the radiation dose–response relationship was taken into account, the residual excess risk for girls was found to be only 5%.

No significant interaction was found between the cumulative adriamycin dose and that of radiation to the heart (Table 7

**Table 7:** Frequency of cardiac abnormality in 229 pts according to the cumulative dose of adriamycin and to the average radiation dose received to the heart

**Parameters**	**Cardiac abnormality^a^**		
**Cumulative dose of adriamycin and average radiation dose to the heart**	**% (nb/N)**	**Relative risk^b^ and 95% CI**	***P*-value**
*<250 mg of adriamycin*			
<5 Gy to the heart	11% (4/32)	1^c^	
⩾5 Gy to the heart	44% (8/18)	4.9 (1.3–18.0)	0.02
*⩾250 mg of adriamycin*			
<5 Gy to the heart	38% (53/141)	5.1 (1.8–14.5)	0.003
⩾5 Gy to the heart	71% (24/34)	6.6 (2.1–20.6)	0.001

a At least one of the following criteria: cardiac failure, FS<25, ES<50, or ESWS>100.

b Adjusted for age at treatment of first cancer, and sex, and stratified on the site of the cancer.

c Reference category.

). The relative risk for a patient who had received both adriamycin therapy and radiation was found to be not significantly different from the product of the risks for each of these risk factors (*P*-value for interaction=0.9).

## DISCUSSION

The aim of the present study was to determine the long-term risk of cardiac disease after adriamycin therapy for a cancer in childhood and the influence of other drugs and radiotherapy on this risk. Our study failed to show a threshold for adriamycin-induced cardiac toxicity, which clearly may occur after less than 250 mg m^−2^. We also failed to show any increased risk associated with drugs considered noxious for cardiac or pulmonary function, such as cyclophosphamide, ifosfamide, and bleomycin. We evidenced a dose–response relationship for the radiation dose to the heart, which was stronger among girls than among boys. According to our results, higher sensitivity to radiation therapy could account for the higher risk observed among girls in some studies. Another important result was the very high risk of long-term cardiac toxicity associated with high-dose adriamycin therapy combined with radiation to the heart.

Among the 447 pts initially targeted in our roster, 218 patients (49%) were not included. This could be the over-riding potential source of bias in our study. These patients were more frequently male and older than those who were known to have developed cardiac failure or who were investigated. They had also received a slightly higher dose of adriamycin and a lower dose of radiation to the heart, but these differences disappeared after adjustment on age. Seven of these patients died from an unknown cause (*n*=1) or causes nonlinked to cardiac disease. This selection strongly limits our ability to assess the absolute value of the incidence of cardiac failures and cardiac abnormality. On the contrary, it should have little influence on our estimation of the relative risk of pathologies associated with treatment by adriamycin and radiotherapy. Indeed, when formulating the hypothesis, where none of the 218 nonincluded patients experienced such pathologies and analyses on was performed the whole cohort of 447 patients, relative risk estimations were extremely similar to those obtained on the 229 included patients.

The major findings of well-conducted studies were listed by Kremer *et al* (2002). Unlike the results reported by Lipshultz *et al* (1991, 1995) and Green *et al* (2001), we did not identify the female sex as a significant independent risk factor for abnormal cardiac function or cardiac failure, nor as a modifying factor of the dose–response relationship between the adriamycin dose and the risk of such disorders. Although the number of cardiac abnormalities (*n*=89) and the range of doses were considerable in our study, our results are not incompatible with the likelihood that female patients may be more vulnerable to adriamycin treatment. Nevertheless, about 80% of the nonsignificant excess risk we observed among girls was explained by a higher risk for a given dose of radiation to the heart (*P*=0.02) and not by a higher sensitivity to adriamycin therapy (*P*=0.8).

In agreement with some studies (Lipshultz *et al*, 1991), but not with others (Silber *et al*, 1993; Kremer *et al*, 2002), a younger age at treatment was found to be a risk factor for both cardiac failure and abnormalities. The age distribution in our series is wider than in the first publication by Lipshultz *et al* (1991). In our opinion, this age factor may not have been investigated adequately in that study because the age range is far narrower and lower in acute leukaemia than in solid tumours in children. However, the occurrence of cardiac abnormalities probably increases with a younger age at adriamycin treatment and with an older age at cardiac evaluation, which are inversely correlated. In some studies, these two parameters are very closely associated and therefore cannot be investigated adequately.

In our series, we included all types of solid tumours, whereas other studies included only lymphoblastic leukaemias (Sallan and Clavell, 1984; Lipshultz *et al*, 1991; Sorensen *et al*, 1997; Nysom *et al*, 1998), osteosarcoma (Lipshultz *et al*, 1995), or Wilms' tumour (Sorensen *et al*, 1995; Green *et al*, 2001). When the two former types were excluded (*n*=71), our results remained unmodified.

We demonstrated the role of the radiation dose to the heart. This result was suggested in some studies (Steinherz *et al*, 1991, 1993, 1995), where radiotherapy (RT) *vs* no RT was studied. A total of 120 patients investigated had received radiotherapy, at some site in the body. During radiotherapy, the radiation dose to the heart is due to primary and scattered radiation if the heart is inside the beam field, and to scattered radiation only, if the heart is outside the target volume. Even if the heart is less than a tenth of a cm from the beams, the scattered radiation received outside the beam field is not, however, negligible. We estimated the radiation dose received at 6 points in the heart. An average dose of 5 Gy or more had been delivered to the heart in 52 pts: an increased risk of cardiac disease was demonstrated above 5 Gy in this study. The other 68 pts who had received less than 5 Gy were not at increased risk. The role of radiotherapy is well-known (Pai and Nahata, 2000; Green *et al*, 2001; Kremer *et al*, 2002), but no dose–response relationship has been demonstrated.

For adriamycin doses higher than 150 mg m^−2^, we failed to evidence a threshold in the dose–response relationship between adriamycin therapy and both cardiac failure and all cardiac abnormalities in general. This is in agreement with the report of a study limited to the shortening fraction (Bu'Lock *et al*, 1995). Another study was not able to investigate the dose–response relationship for doses lower than 250 mg m^−2^ with sufficient statistical power because most patients had received more than this dose (Lipshultz *et al*, 1991, 1995). However, in Green's case–control study (Green *et al*, 2001), the odds-ratio (OR) for a dose ranging from 200 to 300 mg m^−2^ was 1.1 (95% Cl: 0.3–51), which is lower than our OR of 2.0 for doses from 150 to 250 mg m^−2^ and 4.0 for doses from 250 to 400 mg m^−2^. A difference in the duration of follow-up cannot explain this discrepancy. Owing to the standardisation of the protocols used before 1982, we were unable to investigate the role of the cumulative dose and that of the dose per injection separately, as of great interest in several studies (Von Hoff *et al*, 1979; Sallan and Clavell, 1984; Lipshultz *et al*, 1995). Practically, all our patients had received a 60 mg m^−2^ dose as a bolus injection every 3 weeks.

Like other authors, we found that the risk of cardiac failure increased regularly with time since adriamycin therapy (Figure 1), with no suggestion of a plateau.

Since we found no evidence suggesting an interaction between adriamycin and the effect of radiation on the risk of cardiac failure, we estimated the risk for patients who had received both treatments, not significantly different from the product of the risks for each treatment. Consequently, patients who had received high doses of each treatment had to be considered particularly at risk of cardiac failure.

## CONCLUSION

An increased incidence of cardiac failure and severe cardiac abnormalities with long-term follow-up seen in our study is in accordance with previously published results. The major added cause for concern is the continuous increase in the occurrence of cardiac failure with time, with no sign of a plateau from exposure up to 15 and 25 years of follow-up. In our series, after an average follow-up of 18 years, 39% of the children had a severe cardiac dysfunction or major ventricular overload conditions. As the usual age for the occurrence of cardiac failure in the general population starts from 50 years onwards, this increased incidence of cardiac disorders in cancer survivors who were exposed to adriamycin during childhood could be compounded as they approach the age of 50 years. The higher risk of cardiac failure and abnormalities for the female gender observed in other studies, was found in our study to be essentially due to the higher sensitivity of girls to radiation therapy rather than to a higher sensitivity to the cumulative adriamycin dose in itself.
